# Intracellular Responses Triggered by Cold Atmospheric Plasma and Plasma-Activated Media in Cancer Cells

**DOI:** 10.3390/molecules26051336

**Published:** 2021-03-02

**Authors:** Helena Motaln, Nina Recek, Boris Rogelj

**Affiliations:** 1 Department of Biotechnology, Jozef Stefan Institute, SI-1000 Ljubljana, Slovenia; boris.rogelj@ijs.si; 2 Department of Surface Engineering, Jozef Stefan Institute, SI-1000 Ljubljana, Slovenia; nina.recek@ijs.si; 3 Biomedical Research Institute BRIS, SI-1000 Ljubljana, Slovenia; 4 Faculty of Chemistry and Chemical Technology, University of Ljubljana, SI-1000 Ljubljana, Slovenia

**Keywords:** cold atmospheric plasma (CAP), reactive oxygen species, cell signaling, cell death

## Abstract

Cold atmospheric plasma (CAP), an ionized gas operating at room temperature, has been increasingly studied with respect to its potential use in medicine, where its beneficial effects on tumor reduction in oncology have been demonstrated. This review discusses the cellular changes appearing in cell membranes, cytoplasm, various organelles, and DNA content upon cells’ direct or indirect exposure to CAP or CAP-activated media/solutions (PAM), respectively. In addition, the CAP/PAM impact on the main cellular processes of proliferation, migration, protein degradation and various forms of cell death is addressed, especially in light of CAP use in the oncology field of plasma medicine.

## 1. Introduction

Cold atmospheric plasma (CAP) is partially ionized gas, produced at atmospheric pressure and operating at room/body temperature. Roughly a decade ago, CAP started to be considered for medical therapy despite paucity of supporting biomedical and mechanistic redox chemistry research [1,2]. Most reactive species found in plasma source are known in biology for inter- and intra-cellular communication (redox signalling). Mammalian cells are equipped to interpret the plasma derived redox signals, their composition, strength, and duration, in either cell endurance/fitness or cell death promoting ways. In vitro and in vivo, CAP was shown to act anti-inflammatory, tissue-stimulating, blood flow-enhancing, and would healing on one side [3], and bactericidal, proapoptotic, and anti-tumorigenic [1,4,5] on the other side. CAP’s anticancer capacity led to establishing a new field in medicine called “plasma oncology” [6]. In this field, plasma gained attention owing to its ability to induce cancer cell death [4] and significantly reduce tumour size in mice, without damaging normal cells [7]. In this review, we shall focus on the processes triggered in cancer cells, and leading to their cell death and elimination. In particular, the mechanisms and processes triggered by CAP-origin reactive species, the rise of intracellular reactive oxygen and nitrogen species (RONS), DNA and mitochondria damage, and deregulated expression of survival inhibiting and death promoting genes will be discussed.

## 2. Cold Atmospheric Plasma Devices

Chemically CAP comprises unique mixes of active species such as RONS generated by working gas [8]. Several different gases are currently used to produce CAP such as Helium, Argon, Nitrogen, Oxygen, air, and their mixing; and specific methods and devices for their production were developed, for use in different biomedical conditions [4]. For direct and indirect treatments in medical applications, plasma jet and dielectric barrier discharge-DBD plasma sources appear suitable. Majority of plasma laboratories around the world use tuned/home-made plasma devices [9,10,11]. They are inexpensive and simple to make, but the main drawback of using these home-made devices is that comparison of the results obtained at exactly the same conditions is very difficult, almost impossible. The standardization of the cold atmospheric plasma devices has been attempted to achieve for some time now, but this topic is still subjected to discussion. Though specific devices have been developed and utilized by various research groups, there are some—the DBD device PlasmaDerm^®^ VU-2010 (CINOGY Technologies GmbH), the atmospheric pressure plasma jet (APPJ) kINPen^®^ MED (INP Greifswald/neoplasm tools GmbH), and the SteriPlas (Adtec Ltd., London, UK)—that have been CE-certified as a medical product [4]. In this respect, Jet treatment proved superior to DBD in yielding much stronger cellular response [5]. For this reason, the so-called COST jet, being introduced by a European COST initiative as a reference device, using both reference conditions as well as conditions adjusted to kINPen gas mixtures [12] were evaluated. In these, thiol oxidation revealed dominant under all tested conditions, whereas an Ar + N_2_/O_2_ gas compositions combined with a nitrogen curtain were shown to foster nitric oxide deposition and desired generation of S-nitrosocysteine [12]. This highlights the potential of plasma effects tuning, not only by gas admixtures alone but also by adjusting the surrounding atmosphere.

## 3. Effects of Plasma-Activated Liquids

In most CAP treatment experiments, CAP-derived chemical and physical particles reach the cells via their surrounding milieu. Several studies revealed that final outcome of CAP-induced cytotoxicity does not depend solely on device type, gas mixture, and treatment duration [13,14], but also on the type of the surrounding milieu. This proved true for indirect CAP treatment of seven human cell lines (cancerous: A549, U87, A375, and Malme-3M; non-cancerous: BEAS-2B, HA, and HEMa) with five different plasma-activated media (PAM) (DMEM, RPMI1640, AM, BEGM, and DCBM). Considerable differences were noted in these experiments, whereas direct CAP treatment proved less affected by analysed parameters [15]. Toxicity of PAM in cancer cells could be modulated by controlling the composition of solution (PBS vs. DMEM) [16,17]. CAP-treated PBS seems to inhibit cell growth in a treatment time-dependent manner, showing a linear correlation to the solution peroxide concentration. In contrast, CAP-treated foetal bovine serum (FBS), acting as a model for complex bio-fluids, shows not only cytotoxic effects but also exhibits increased mutagenic potential [18]. Compared to water, a higher production of reactive species (H_2_O_2_ and NO_2_^−^) can be detected in 2% gelatine polymer solution after CAP treatment, with RONS amounts generated in up to 12-times higher concentration, thus resulting in its higher efficiency to kill U-2 OS bone cancer cells [19]. Likewise, CAP treated DMEM shows increased effect on proliferation and apoptosis of A431 and HaCaT cutaneous squamous carcinoma cells, compared to CAP treated PBS, though intracellular ROS levels are more increased in the latter [20]. Among RONS that are produced in these CAP-activated liquids, hydrogen peroxide, nitrite, and nitrate appear to be mainly responsible for cytotoxic and genotoxic effects. CAP-PBS appears more efficient than 0.9% CAP-NaCl due to more extensive RONS production [21]. In addition to the mentioned CAP-activated liquids, a higher production of RONS was also observed in more polymer solutions such as CAP-treated alginate (in solution/non-crosslinked phase), and shown to affect cells in vitro [22]. Additionally, the anti-cancer potential or plasma-conditioned liquids for in vivo therapies has been thoroughly discussed by Sole-Marti et al. They claim plasma-activated liquids represent an alternative to direct CAP treatment [23,24,25], because they may allow treatment of malignant tumours located in inner organs of the body, by means of an injection, thus avoiding multiple surgeries [23]. Further studies are needed to determine the nature, causes, and effects of the cyto- and genotoxic potential of solutions exposed to plasma discharges and generating oxidative stress in cells to ensure the long-term safety of novel plasma applications in medicine and healthcare.

## 4. Plasma’s Ability to Differentially Affect Cell Fitness

CAP treatment promotes cell growth or cell death depending on the cell type, plasma type, and exposure parameters [26,27]. The exposure parameters effects on these processes were presented already by Bauer [28] and Tanaka [29]. The general consensus is that the exposure of living cells to the CAP or PAMs initiates their cellular response, mainly due to the oxidative stress signals generated [30,31]. The cells’ early response to this oxidative stress signals very much resembles the initial response of the cells to transient stress, which is reversible and allows cells to resume normal cell functions or even boost their metabolism after stress withdrawal. This way CAP was shown to induce stress granule formation in the exposed cells via eIF2α-signalling, and in dynamic very much resembling stress granule formation in cells exposed to Arsenite—the oxidative stress inducer [26]. Stress granules are transient structures within the cells formed by proteins and RNA, that initiate translation halt, and protect the cellular proteins and RNA during stress. These structures also reversibly disassemble upon stress signal withdrawal to enable metabolic boost in recuperating cells, possibly very much utilised in wound healing [3]. However, during long-lasting stress conditions, cell response triggers and activates the downstream pathways leading to cell death—the process utilised in plasma oncology. CAP irradiation was shown to reduce lung adenocarcinoma cell viability [32] and to induce cell death in colon cancer, melanoma, cervical cancer, glioma, multiple myeloma, and many more [5,33]. The selective toxicity of breast cancer cells over the normal mesenchymal stem cells (MSCs) was very early revealed [34]. Still, to eradicate the cells, in other words to force the cell to succumb to cell death, several mechanisms in different cellular compartments must get activated.

## 5. Intercellular Effects of Reactive Species Generated by CAP

Among the plethora of reactive oxygen and nitrogen species (RONS) produced in plasma-activated saline solutions and buffers, hydrogen peroxide, nitrite, and nitrate appear most represented and responsible for cytotoxic and genotoxic effects [21]. The formation of ^•^OH radicals generated by CAP depends on the type of rare gas used, the yield of production of ^•^OH and correlates inversely with ionization energy in the order of krypton > argon = neon > helium [35]. The electron paramagnetic resonance (EPR) spectra analyses of aqueous solutions exposed to Ar-CAP revealed the formation of enormous amounts of ^•^OH radicals, with small amounts of H atoms with no nitric oxide or pyrolysis radicals present. Hydrogen peroxide H_2_O_2_, the recombination product of ^•^OH and OCl^−^ is the most likely formed reactive oxygen species [35] and is speculated to be the one toxic trigger, particularly of cancer cells response [36]. Recently, intracellular signalling cascades have been reviewed and schematically presented elsewhere [9,37], yet here we shall focus on them, regarding their sequential activation in different subcellular compartments.

### 5.1. Reactive Species Interact with Cell Membrane Enzymes

During cell/tissue treatment with CAP or PAM, cellular and organelles membranes represent the natural interphase, which first comes in contact with the above mentioned RONS produced in PAM or within the cells. The membranes allow for the translation of RONS chemical reactivity into distinct biological responses [38]. Tumour cells are protected against intercellular apoptosis by inducing signalling with increased expression of membrane-associated enzymes catalase and superoxide dismutases [39]. None of the long-lived species found in PAM, such as nitrite and H_2_O_2_, nor OCl^−^ or NO seem to have the potential to interfere with catalase-dependent control of apoptotic cell death-inducing signalling within tumour cells when acting alone. However, these reactive species acquire this potential when involved in a sequential multi-step process. The first step involves the formation of primary singlet oxygen (^1^O_2_) through the complex interaction between NO_2_^−^ and H_2_O_2_ [40]. ^1^O_2_ then inactivates some membrane-associated catalase molecules on at least a few tumour cells. Consequently, H_2_O_2_ and peroxynitrite that are produced continuously by tumour cells [41], and are usually decomposed by their protective membrane-associated catalase, are found to survive at the site of locally inactivated catalase [42]. With some protective catalase molecules inactivated in these tumour cells, the surviving cell-derived, extracellular H_2_O_2_ and ONOO^−^ form secondary ^1^O_2_ [42,43]. These continue to inactivate catalase on the triggered cells and adjacent cells via autoamplificatory propagation of the secondary singlet oxygen. The bystander effect on signalling between treated and untreated tumour cells (possibly within tumours) depends on the generation of secondary singlet oxygen by the treated cells and singlet oxygen-mediated catalase inactivation of the untreated recipient cells [42,44]. CAP and PAM-derived reactive species are merely the trigger for the activation of autoamplificatory mechanisms of tumour cells. The exposure to CAP or PAM initially inactivates only a small percentage of protective membrane-associated catalase molecules in the tumour cells [28]. Then the tumour cells efficiently propagate their cell death through their own CAP-induced RONS signalling [38,44].

At the inactivated catalase site, CAP and cell-generated H_2_O_2_ enters the cell via aquaporins, leading to intracellular glutathione depletion [42], since cysteine is the main target of effective ROS [36]. This abrogates the cell protection towards lipid peroxidation and sensitises the cells for apoptosis induction [28,42]. Optimal inactivation of catalase thus seems to allow for efficient cell-death induction through the NADPH oxidases 1 (NOX1) signalling pathway driven by HOCl, the signalling that is on cell membranes finalized by lipid peroxidation [28]. Accordingly, CAP was shown to induce increased lipid peroxidation and nitric oxide production in B16 melanoma cells compared to non-malignant L929 cells [33]. Though the above-mentioned experimentally established model based on a triggering function of CAP and PAM-derived H_2_O_2_/nitrite sufficiently explains selective cell death in tumour cells, also based on their own RONS [42], surprisingly a recently published mathematical model [45] claimed that catalase-dependent activation of the apoptotic/cell death pathways is unlikely to contribute to the observed anti-cancer effect of CAP.

### 5.2. CAP Affects Membrane Integrity, Permeability, and Endocytosis

The changes in the cell membranes induced by CAP and PAM inevitably affect their normal functions. Computer simulations confirmed CAP/PAM-oxidizes a phospholipid bilayer to exhibit a decrease of the free energy barrier for translocation of various substances, including melittin, when compared to the non-oxidized bilayer [46]. CAP treatment was shown to enhance translocation of low molecular weight (ATP), as well as molecules, sized up to 150 kDa, through the cytoplasmic membrane [47,48,49]. PAM efficiency herein reveals cell type dependency (efficiency proved in HeLa cells vs. none in 4T1 cells) [47]. The detection of non-membrane-permeable fluorescein diacetate and endogenously synthesized ATP confirmed increased membrane permeability in human osteosarcoma (U-2 OS, MNNG-HOS) [48,49] and U373MG glioblastoma cells [8]. CAP/PAM treated cell membranes rich in peroxidised lipids are trafficked into the cells via membrane repairing endocytosis. Their enhanced uptake is clathrin-dependent with the formation of lysosome directed vesicles [8,50]. CAP-stimulated membrane repair via increased endocytosis can accelerate the uptake of dextran and several nanoparticles [48,49]. Besides, CAP-induced changes in the cell membrane of U-2 OS, MNNG/HOS, A673, and RD-ES cells also afflict their cytoskeleton composition and G/F actin distribution [48,51], leading to the formation of actin stress fibres [47]. A model, based on the expression of aquaporins, was proposed to explain why cancer cells respond to CAP treatment with a more significant rise in ROS than normal cells. Namely, cancer cells express more aquaporins on their cytoplasmic membranes, which causes the H_2_O_2_ uptake speed in cancer cells to be faster than that in normal cells, resulting in faster cancer cell killing. Due to membrane changes, glioblastoma cells indeed consume H_2_O_2_ much faster than do astrocytes after CAP/PAM treatment [52], which supports the selective model based on aquaporins.

### 5.3. Changed Ionic Fluxes and pH Affect Mitochondria and Endoplasmic Reticulum

The abnormalities in membrane transport highly affect the intracellular conditions in the cytoplasm and function of various organelles. CAP was shown to destroy the ultrastructure of HepG2, A549, and HeLa cells to different degrees, demonstrated in perturbed ionic fluxes, nuclear fragmentation, and organelle damage [53]. Increased intracellular ROS concentration in He-CAP treated cells was shown to reduce the intracellular pH [54]. Both intracellular ROS and pH affect Ca^2+^ fluxes. CAP induces increased Ca^2+^ influx in melanoma cells in acidic pH than in physiological conditions [55]. Since CAP-induced cytoplasmic Ca^2+^ increase occurs in melanoma cells even in the absence of extracellular calcium, this indicates the Ca^2+^ increase to originate from intracellular stores. In this respect, ryanodine and cyclosporin A analyses confirmed the involvement of the endoplasmic reticulum and the mitochondria [56].

Intracellular NO formation induced by CAP treatment is also pH-dependent, with enhanced protein nitration occurring under acidic conditions. The pH and RNS affect the ion pumps, mitochondrial membrane permeability, and mitochondrial membrane potential [57]. CAP modifies the dynamics of intramitochondrial H_2_O_2_ and superoxide anions, i.e., the rhythm and shape of ROS oscillation are disturbed by H_2_O_2_ infusion [58]. The present computational model demonstrates that CAPs crucially affect essential mitochondrial functions, which in turn affect intracellular redox signalling, metabolic cooperation, and cell fate decision on survival or death induction. CAPs control the ROS oscillatory behaviour, nicotinamide adenine dinucleotide redox state and ATP/ADP conversion through the respiratory chain, the TCA cycle, and intracellular ROS regulation system [58]. Moreover, CAP treatment decreases the glutathione (GSH) levels in cells and results in the loss of mitochondrial membrane potential and cytochrome c release, leading to cell death. Pre-treating the cells with an antioxidant *N*-acetyl-l-cysteine (NAC) dramatically decreases the death of CAP-treated cells [54]. Disruption of mitochondrial membrane integrity in CAP treated cells [57] results in decreased ATP production and downregulation of survival PI3K/AKT/mTOR and RAS/MEK pathways [59]. Likewise, CAP induced Nrf2-mediated oxidative endoplasmic reticulum stress response, PPAR-alpha/RXR activation, and excessive peroxisomes production in the treated cells [60].

### 5.4. CAP Treatment Affects Nuclear DNA Content and Replicative/Transcriptional Activity

The CAP generated stress stimuli entering the nuclei directly via cell membrane or organelle signalling pathways, showing the effect on DNA content and processes of DNA replication and gene expression, preceding pro- and anti-survival pathways activation. CAP treatment induces DNA damage and promotes induction of Sub-G1 phase stop in melanoma cells [61]. Likewise, incubation of cholangiocarcinoma cells BPH-1 and PC-3 cells with PAM leads to double-strand DNA breaks [62], which are also detected by histone H2AX phosphorylation in the outermost layers of 3D adenocarcinoma cell spheroids upon PAM treatment [63]. As DNA damage can be avoided by catalase addition, this points to H_2_O_2_ as a major player in observed PAM genotoxicity [63,64]. As superoxide dismutase and D-mannitol scavengers can also reduce DNA damage, this indicates O_2_^(−)^ and OH^−^ involvement in H_2_O_2_ formation [63].

In CAP-treated cells, DNA breaks are followed by an increased phosphorylation and activation of the cell cycle master regulators—checkpoint kinases CHK1/2 and mitogen-activated (MAP) kinases, increased expression of MAP kinase signalling effectors (e.g., heat shock protein Hsp27), epithelium-derived growth factors, and cytokines (Interleukins 6 + 8) [65]. In a human skin cell model, CAP causes the phosphorylation of serine- (ATM) and serine/threonine-protein kinase (ATR), where ATM acts as a direct redox sensor yet without relevant contribution to phosphorylation of the histone A2X. This is followed by transient phosphorylation and nuclear translocation of p53 [62,65], phosphorylation of Rad17, Cytochrome c release, and activation of Caspase-3 [61], leading to cell cycle arrest and cell death.

Transcription wise, CAP treatment deregulates the expression of over 934 genes, which cluster into 40 different pathways, with p53 pathway being the most enriched. Surprisingly, many p53 pathway-related genes might be activated by other stimuli, in a p53-independent manner [66]. Likewise, 112 and 843 deregulated genes were detected in CAP-treated U937 and SK-mel-147 cells, respectively. However, only 4 and 2 genes, respectively, were found commonly regulated by H_2_O_2_ and CAP, indicating that non-ROS constituents are responsible for the regulation of the majority of CAP-regulated genes, including both *PTGER3* and *HSPA6* [67]. CAPs also deregulate the expression of several transcription factors, including *c-FOS* [68] and Yes-associated protein YAP/transcriptional enhancer associated domain TEAD [29]. In MCF-7 breast cancer cells, up and downregulation of *ZNRD1* gene (DNA directed RNA-polymerase 1 subunit) correlated with long and short CAP treatment scheme. Its antisense long noncoding RNA, ZNRD1-AS1 was shown to be regulated in the opposite direction and shown to increase the expression of other cis-genes including *PPP1R11* involved in proteasomal degradation [69]. In lung cancer cells, CAP treatment was shown to inhibit cell proliferation by depressing pERK and pAKT downstream signalling [70]. Contrary, the miR-203a expression normally downregulated in lung cancer tissue was increased in the CAP treated cells. Increased miR-203a inhibited proliferation and targeted *BIRC3*, *BIRC5* inhibitors of apoptosis for silencing in lung cancer cells [71].

Besides the direct effect on DNA, CAP also displays an epigenetic effect. In the H3K4me3 MCF-7 breast cancer cell line, CAP treatment changed the methylation level of 899 genes. A histone demethylase JARID1A was induced by CAP via ROS signalling, and was shown to inhibit *HSCB* and *PRPS1* oncogenes expression in breast cancer cells. CAP inhibits cancer cell proliferation by modulating the H3K4 methylation level corresponding to oncogenes [72]. The hypomethylation effect induced by CAP treatment is enhanced in oestrogen-negative MDA-MB-231 cells [73], which indicate that plasma induces epigenetic and cellular changes in a cell type-specific manner.

### 5.5. CAP Affects Cytoplasmic Metabolite Content

Lack of pyruvate is known to increase PAM’s cytotoxic potential in affected cancer and healthy cells by increasing 10–100 times the concentration of present H_2_O_2_ without altering that of nitrites [74]. Contrary, CAP-treated AML cells display changed metabolism of alanine, aspartate, d-glutamine, and d-glutamate. Glutaminase activity decreases after CAP treatment, leading to intracellular glutamine accumulation and leukaemia cells death [75]. In vivo CAP treatment of endothelial cells results in downregulated xantosine and proline metabolites, though KEGG pathway analysis revealed alanine, aspartate, glutamate, and purine metabolism pathways to be most suppressed [76]. Yet, He-CAP treatment of myeloma cells revealed the beta-alanine metabolism pathway to be most changed, followed by propanoate and linoleic acid metabolism [77]. The alanine decrease is also consistent with the metabolomic profiles of U251 cells exposed to the CAP-treated Ringer’s lactate solution, which shows increased generation of acetyl-CoA for lipid metabolism from alanine and asparagine [78]. CAP is also known to modify the amino acids of proteins, affecting the protein structure and function, which results in changes of the secondary and/or tertiary structure of the proteins in the presence and absence of co-solvents, as demonstrated for lysozyme, horseradish peroxidase, myoglobin, α-chymotrypsin, lipase, MTH1180, haemoglobin, and bacteriorhodopsin [5].

## 6. CAP Affects Major Cell Processes

**Figure 1:**
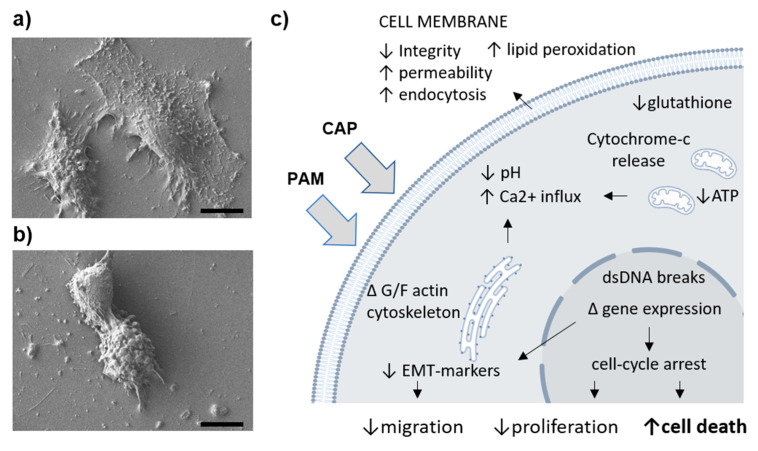
Cold atmospheric plasma poses effects on cellular morphology and various cellular processes. The appearance of (**a**) non-treated and (**b**) plasma treated immortalized fibroblasts showing reduced cell proliferation, cell rounding, and cell death, 24 h upon high dose plasma exposure. (**c**) A schematic overview synthesizing plasma induced (intra)cellular perturbations affecting some major cell processes. Scale bar 10 µm.

All mentioned disruptions of vital cellular components—membranes, cytoplasmic milieu, and organelles—have drastic effects on cell fate. A normally vital cell that is not in the terminally differentiated state would either replicate the genetic material and proliferate or migrate in between the cell cycles. Should such cell face any stress signals, its proliferation and migration will be the first to seize, followed by either cell-death avoiding (autophagy) or cell death promoting processes (see Figure 1).

### 6.1. Proliferation

A strong anti-proliferative effect of CAP/PAM was demonstrated in chondrosarcoma CAL-78, SW1353 [48], A549, H1299 [71], U-2 OS MNNG cells, 3T3 fibroblasts, HaCaT keratinocytes [79], glioblastoma cells [29], pancreatic cancer cells [80], MG63 osteosarcoma cells [57,81], and C2C12 myoblasts [82]. In this respect, CAP-treated osteosarcoma cells exhibit an increase of PRX 1 and ratio change of oxidized to reduced forms of PRX1 and PRX2, with an increased cellular concentration of oxidized dimer. This effect can be attenuated by N-acetylcysteine (NAC), an antioxidant supplement known to suppress redox homeostasis changes [81].

Furthermore, the expression of γH2A.X (pSer139), an oxidative stress reporter indicating S-phase DNA damage described previously is enhanced in CAP treated cells that are in the S phase of the cell cycle [83]. This coincides with the notion that post CAP/PAM treatment, the percentage of cells in the G2/M phase increases and cells show G2/M arrest [82]. Cancer cells are highly proliferative (the highest proportion of the cells in the S-phase), thus CAP treatment was shown to decrease their viability via G2/M arrest in a dose-dependent manner, whereas no such CAP effect was noted in HUVEC and NHA cells [84]. Cell lines differ in their proliferative rates, the reason why CAP treatment substantially shrinks U87-Red spheroids and to a lesser degree, less proliferative U251-Red spheroids [85]. In vivo CAP treatment can decrease glioblastoma tumour volume by 56% and increase mouse life span up to 60% [86].

### 6.2. Migration

CAP treatment inhibits the migration and invasion of BrCa cells [34], whereas CAP-Ar treatment of mammary carcinoma cells (MCF-7, MDA-MB-2311) leads to a complete loss of cellular motility [87]. The D-17 and DSN osteosarcoma cells also exhibited reduced migration and invasion activity when treated with CAP [2]. This is possibly related to a decrease of epithelial-to-mesenchymal-transition (EMT) markers (E-cadherin, YKL-40, N-cadherin, SNAI1) and stem cell (CD133, ABCB5) markers, as observed in CAP-treated melanoma cells. Namely, the expression of these markers describes a highly motile cancer cell phenotype [88]. Lower expression of stem cell markers could account for decreased sphere-formation ability of glioblastoma cells, dependent on the presence of stem-like cells [89]. Yet, possibly, cell-type dependent 3D spheroids of human osteosarcoma cells oppositely demonstrated increased stem cell marker expression upon treatment with CAP activated Ringer’s solution [90]. Still, CAP in conjunction with temozolomide reduces cell migration in glioma cells via increased αvβ3 and αvβ5 cell surface integrins expression, that enhance cell adhesion [91], whereas CAP-suppression of migration ability in myeloma cells proceeds via decrease of MMP-2 and MMP-9 secretion [92], metalloproteinases crucial for extracellular matrix degradation.

However, milder/shorter CAP exposures were noted to promote the motility of human HT-1080 cells observed by extended cell shape, membrane protrusion formation, and increased cell surface area, but not cell death induction, despite the production of intracellular ROS and Ca^2+^ [93]. Similarly, CAP triggered production of nitric oxide (NO) was noted to enhance endothelial cell migration in the angiogenesis model [94].

### 6.3. Autophagy and Proteosomal Degradation

Upon halted proliferation/migration, cells try to cope with stress by increased autophagy and proteasomal degradation of misfolded and aggregated proteins, respectively. Regarding proteasomal protein degradation, the RONS generated by He and He-N_2_ CAPs, are increased in treated human epithelial cells [95], whereas PAM treatment increases autophagic cell death in endometrial cancer cells in a concentration-dependent manner. In PAM-treated cells, the mTOR pathway is inactivated [96]. Autophagy was recorded upon CAP exposure in primary prostate cancer cells, whereas established cancer cell lines exhibited necrosis and apoptosis [64]. The autophagy inhibitor MHY1485 was shown to partially inhibit the autophagic cell death induced by PAM treatment [85]. A huge CAP effect on autophagy was noted in melanoma cells, while there was only a minor effect on autophagy noted in L929 cells [33]. Likewise, CAP-treated glioblastoma cells accumulated acridine orange positive vesicles, indicative of acidic lysosomes, and associated with their concomitant cell death, yet with no increase of autophagy [97].

Autophagy is reported either as a survival or death-promoting pathway and as such remains highly debatable in different kinds of cancer. CAP and silymarin nanoemulsion trigger autophagy in G-361 cells by activating the PI3K/mTOR and EGFR pathways [59]. This blocks downstream survival pathways via reduced *HRAS* and *MEK* genes expression; modulated *ZKSCAN3*, *TFEB*, *FOXO1*, *CRTC2*, and *CREBBP* transcription factors expression, and enhanced *BECN-1*, *AMBRA-1*, *MAP1LC3A* and *SQSTM,* autophagy-related genes expression [59].

### 6.4. Senescence and Cell Death Involving Apoptosis, Necrosis, and Pyroptosis

At the cross-road of cell faith decision—whether to respond to stress with high energy-consuming either protective autophagy or cell death activating mechanisms—some cells can simply duck and enter the low energy consuming state of cellular senescence. This senescence (quiescence) phase enables the cells to re-activate normal cellular metabolism upon stress seizure, provided cell organelles and DNA are not damaged beyond repair. In this respect, low doses of CAP have been shown to induce senescence in melanoma cells, confirmed by a positive H3K9 immunofluorescence, SA-β-Gal staining, and p21 expression [98]. CAP-He treatment of normal human dermal fibroblasts and adipose-derived stromal cells also does not induce cell death but leads to minor DNA damage, proliferation arrest with an increase in p53/p21, p16 expression, characteristic morphological changes, and secretion of pro-inflammatory cytokines defined as the Senescence-Associated Secretory Phenotype (SASP), associated with a glycolytic switch and increased mitochondria number [99].

However, upon CAP treatment, several cancer cell lines including HEI-193 and mouse SC4 VS cells, preferentially exhibit programmed cell death or apoptosis and necrosis [64,100]. The programmed cell death or apoptosis can be triggered by extrinsic (extracellular trigger origin) and intrinsic (intracellular trigger origin) signalling pathways that can overlap at various levels. CAP generated RONS in THP-1 human monocytic leukaemia cells induce apoptotic cell death at lower (1 min exposure) and necrosis at higher dosage treatment (3 min exposure to Ar-CAP, 20kHz low frequency at 18kV with a flow rate 2 L/min) [101]. Similarly, Ar-CAP treatment causes apoptosis of human lymphoma U937 cells, whereas Ar + N_2_-CAP proves to be less efficient [102]. On the other hand, PAM induces apoptosis in triple-negative breast cancers rather than the other subtypes of breast cancer cells, possibly due to genome mutation rate, hyper-activated MAPK/JNK, and NF-kB pathways in the former [103]. MAPK-induced apoptotic signalling was also noted in PAM treated TE354T basal cell carcinoma [104] and A875 melanoma cells, where CAP increased Sestrin2 expression and activated its downstream iNOS, Fas, and p38/MAPK signalling to induce apoptosis via Fas/TRAIL-mediated cell death receptor extrinsic pathway [89,105]. In CAP-treated myeloma cells, p53 proved to be a feedback activator of Fas expression [106]. Downstream caspase-8 activation was observed in CAP-treated Jurkat cells [107]. The levels of cellular glutathione and peroxidases were found to be crucial for CAP induced cell death, showing increased RONS levels to be primary apoptotic triggers [108,109].

Regarding the intrinsic apoptosis pathway also induced by CAP treatment, DNA damage is often described as an intracellular trigger. CAP/PAM treatment increases intracellular ROS, and DNA damage in Jurkat cells [107], which, in CAP treated osteosarcoma cells, leads to increase of p53/phospho-p53 expression, [107,110] and in HT29 and SW480 colon cancer cells to increase of p21 expression [111], resulting in cell cycle blockage and apoptosis. DNA lesions inducing apoptosis are in CAP/PAM treated cancer cells accompanied by 8-oxoguanine(8-oxoG) formation [62], up-regulated 8-oxoG repair enzyme [112], and elevation of DNA-damage inducible protein GADD45 α [29]. The CAP/PAM induced DNA damage induces apoptosis by activation of downstream signalling pathways involving ASK1 stimulation in G-361 melanoma cells [27] and c-JUN/AKT/AMPK or STAT3 in U-2 OS cells [74]. The p53, which is involved in both extrinsic and intrinsic apoptotic signalling, was shown to suppress the expression of Bcl-2 and XRCC1 and increases that of Bax protein resulting in apoptosis and inhibited DNA repair in CAP treated cancer cells [53,107,108,109]. Both extrinsic and intrinsic apoptotic pathways merge at the level of death, executing enzymes—caspases 3 and 7. CAP treatment causes massive caspase 3/7 activation, cleavage, and morphological changes of cell architecture in prostate cancer LNCaP cells [108,109], cholangiocarcinoma cells [62], human endothelial cells HDMEC [113], and G-361 human melanoma cells. This is accompanied by increased PARP level and a blocked HGF/c-MET pathway [88]. As cell death in CAP-treated cells could not be abrogated entirely by pan-caspase and receptor-interacting serine/threonine-protein kinase 1 (RIK1) inhibitors [100], this points toward the involvement of other cell death-inducing cascades.

### 6.5. Immune Response Activating Cell Death

In contrast to apoptosis—cell death without any inflammatory outcome—cell death mechanisms that inherently result in inflammation are pyroptosis and immunogenic cell death (ICD). Recently, CAP was shown to induce pyroptosis, another highly inflammatory programmed cell death, via ROS generation in gasdermin E expressing tumour cell lines [114]. The basal level of gasdermin E protein positively correlates with the cell’s sensitivity to CAP-induced pyroptosis, which depends on the activation of mitochondrial pathways (JNK/cytochrome c/caspase-9/caspase-3) and the cleavage of gasdermin E [114]. ICD, on the other hand, involves changes in the composition of the cell surface, as well as the release of soluble mediators, which operate on a series of receptors expressed by dendritic cells, to stimulate the presentation of tumour antigens to T cells and elicit tumour cell death [115]. ICD is mediated by the release of damage-associated molecular patterns (DAMPs). These molecules are normally retained within cells and integrated into their normal functioning, but once released outside the cells, they act as danger signals. The list of DAMPs includes calreticulin, heat shock proteins (HSPs) 70 and 90, high-mobility group box 1 (HMGB1), ATP, annexin A1, type I interferons (IFNs), mitochondrial DNA, and many other [116]. In this way, PAM rich in H_2_O_2_ was shown to increase calreticulin exposure and ATP release in tumour cells [117]. CAP exposure induced cell death of MX-7 rhabdomyosarcoma cells [118] and of vestibular schwannoma cancer cells [119] via similar calreticulin and HSP70 externalization and increased HMGB1 release. Prostate cancer cells exposure to CAP-PBS resulted in their enhanced immunostimulatory secretion profile (higher TNF-α and IFN-γ, lower TGF-β), and increased phagocytosis by dendritic cells [17], whereas in CAP-exposed keratinocytes, expression of key regulators important for inflammation (IL-8, TGF-β1, and TGF-β2) was found increased [120]. The cellular processes addressed above together with de-regulated genes in cells exposed directly to CAP or to CAP-activated media and other CAP-activated solutions are summarized in Table 1.

## 7. Conclusions

In summary, further insight into specific interactions of plasma-derives species with biological cell/tissue, as well as with subcellular systems (membranous and non-membranous organelles), is highly desired, to enable the fine-tuning of the treatment condition potentially utilized in plasma medicine. Many studies have stressed that improving plasma source and design, and allowing for standardization of protocols and procedures is of utmost importance. An elevated number of proposed clinical trials is expected, though exact treatment regimes, media compositions, and cell type specificity for the desired effect still need to be defined. Regarding the intracellular putative autoamplificatory and positive or negative feedback loops regulated by high or low dose CAP treatment, more detailed knowledge still remains to be provided. All these further studies are warranted to determine the nature, causes, and effects of the cyto- and genotoxic potential of solutions and media exposed to various forms of CAPs to ensure the long-term safety of novel plasma applications in medicine.

## Figures and Tables

**Table 1:** Summary of cellular processes and genes either upregulated (↑) or downregulated (↓) in different cells exposed to CAP and CAP-activated liquids.

Med. Type	Cell Type	Process Affected	Deregulated Genes	Ref.
CAP-media	CAL-78, SW1353, A549, H1299, U-2 OS, 3T3 fibroblasts, HaCaT keratinocytes, glioblastoma cells, Pancreatic cancer cells, C2C12 myoblasts	↓ proliferation	↑ *PRX1, PRX2*	[29,48,71,79,81,82]
CAP-Ringer’s solution	MG-63 osteosarcoma cells	↓ proliferation		[57]
CAP-media	MDA-MB-231, BrCa, DN-17, DSN osteosarcoma cells, MCF-7	↓ migration	-	[2,34,87]
CAP-media	Melanoma cells	↓ migration	↓ *E-cadherin, YKL40, N-cadherin*, *SNAI1*	[88]
CAP-Ringer’s solution	MG-63 osteosarcoma cells (3D)	↑ migration ↑ adhesion	↑ *MMP2, MMP9* ↑ *FN1, PTK2*	[90]
CAP-media + Tmz	Glioma cells	↓ migration	↑ *αvβ3, αvβ5*	[91]
CAP-media	Myeloma cells	↓ migration	↑ *MMP2, MMP9*	[92]
CAP-media	Melanoma cells, glioblastoma cells (3D), MG-63 osteosarcoma cells (3D)	↓ stemness ↑ stemness	↓ *CD133, ABCB5* ↑ *BGLAP, ALPL, BMP2, RUNX2*	[88,89,90]
CAP-media	Human epithelial cells, primary prostate cancer cells, melanoma cells	↑ autophagy	-	[33,64,95]
CAP-media	Endometrial cancer cells	↑ autophagy	↓ *mTOR, PI3K*	[96]
CAP-media + silymarin	G-361 cells	↑ autophagy	↓ *HRAS, MEK* ↑ *BECN1, AMBRA1, MAP1LC3A, SQSTM*	[59]
CAP-media	Melanoma cells, dermal fibroblast, adipose-derived stromal cells	↑ senescence	↑ *H3K9, p21* ↑ *p53, p16*	[98,99]
CAP-media	HEI-193, mSC4 VS, THP-1, U37	↑ necrosis ↑ apoptosis	-	[64,100,101,102]
CAP	T-lymphoblastoid leukemia cells, LNCaP prostate cancer cells	↑ apoptosis	↑ *p53, Bax* ↓ *Bcl2, XRCC1*	[53,107,108,109]
CAP-media	BrCa cells, TE354T basal cell carcinoma, A875 melanoma cells, G361 melanoma cells, U-2 OS	↑ apoptosis	↑ *MAPK, JNK, NFkB* ↑ *Sestrin2, p38, MAPK, Fas* ↑ *Ask1, cJUN, STAT3*	[27,74,89,103,104,105]
CAP-media	Myeloma cells, osteosarcoma cells, HT29, SW480 colon cancer cells	↑ apoptosis	↑ *p53, Fas* ↑ *p21, OGG1, GADD45*	[29,62,106,107,110,111,112]
CAP-media	Prostate cancer LNCaP, choloangiocarcinoma cells, HDMEC, G-361	↑ apoptosis	↑ *PARP* ↑ *Casp3, Casp7*	[62,88,108,109,113]
CAP-media	MX-7, vestibular schwannoma cancer cells	↑ ICD ↑ apoptosis	↑ *CALR* ↑ *HMGB1, HSP70*	[118,119]
CAP-PBS	Prostate cancer cells Kertinocytes	↑ ICD ↑ apoptosis	↑ TNF-α, IFN-γ, ↓ TGF-β ↑ IL-8, TGF-β1, TGF-β2	[17,120]

## Data Availability

Not applicable.

## References

[1] Privat-Maldonado A., Schmidt A., Lin A., Weltmann K.D., Wende K., Bogaerts A., Bekeschus S. (2019). ROS from physical plasmas: Redox chemistry for biomedical therapy. Oxidative Med. Cell. Longev..

[2] Lee J., Moon H., Ku B., Lee K., Hwang C.Y., Baek S.J. (2020). Anticancer effects of cold atmospheric plasma in canine osteosarcoma Cells. Int. J. Mol. Sci..

[3] Lou B.S., Hsieh J.H., Chen C.M., Hou C.W., Wu H.Y., Chou P.Y., Lai C.H., Lee J.W. (2020). Helium/argon-generated cold atmospheric plasma facilitates cutaneous wound healing. Front. Bioeng. Biotechnol..

[4] Bernhardt T., Semmler M.L., Schäfer M., Bekeschus S., Emmert S., Boeckmann L. (2019). Plasma medicine: Applications of cold atmospheric pressure plasma in dermatology. Oxidative Med. Cell. Longev..

[5] Attri P., Park J.H., De Backer J., Kim M., Yun J.H., Heo Y., Dewilde S., Shiratani M., Choi E.H., Lee W. (2020). Structural modification of NADPH oxidase activator (Noxa 1) by oxidative stress: An experimental and computational study. Int. J. Biol. Macromol..

[6] Gümbel D., Bekeschus S., Gelbrich N., Napp M., Ekkernkamp A., Kramer A., Stope M.B. (2017). Cold atmospheric plasma in the treatment of osteosarcoma. Int. J. Mol. Sci..

[7] Keidar M., Walk R., Shashurin A., Srinivasan P., Sandler A., Dasgupta S., Ravi R., Guerrero-Preston R., Trink B. (2011). Cold plasma selectivity and the possibility of a paradigm shift in cancer therapy. Br. J. Cancer.

[8] He Z., Liu K., Scally L., Manaloto E., Gunes S., Ng S.W., Maher M., Tiwari B., Byrne H.J., Bourke P. (2020). Cold Atmospheric plasma stimulates clathrin-dependent endocytosis to repair oxidised membrane and enhance uptake of nanomaterial in glioblastoma multiforme cells. Sci. Rep..

[9] Yan D., Sherman J.H., Keidar M. (2017). Cold atmospheric plasma, a novel promising anti-cancer treatment modality. Oncotarget.

[10] Zaplotnik R., Kregar Z., Biščan M., Vesel A., Cvelbar U., Mozetič M., Milološevič S. (2014). Multiple vs. single harmonics AC-driven atmospheric plasma jet. EPL Europhys. Lett..

[11] Zhou R., Zhou R., Zhuang J., Zong Z., Zhang X., Liu D., Bazaka K., Ostrikov K. (2016). Interaction of atmospheric-pressure air microplasmas with amino acids as fundamental processes in aqueous solution. PLoS ONE.

[12] Lackmann J.W., Bruno G., Jablonowski H., Kogelheide F., Offerhaus B., Held J., von der Gathen V.S., Stapelmann K., von Woedtke T., Wende K. (2019). Nitrosylation vs. oxidation—How to modulate cold physical plasmas for biological applications. PLoS ONE.

[13] Mokhtari H., Farahmand L., Yaserian K., Jalili N., Majidzadeh-A K. (2019). The antiproliferative effects of cold atmospheric plasma-activated media on different cancer cell lines, the implication of ozone as a possible underlying mechanism. J. Cell. Physiol..

[14] Attri P., Park J.H., Ali A., Choi E.H. (2018). How does plasma activated media treatment differ from direct cold plasma treatment?. Anti Cancer Agents Med. Chem..

[15] Biscop E., Lin A., Van Boxem W., Van Loenhout J., De Backer J., Deben C., Dewilde S., Smits E., Bogaerts A. (2019). Influence of cell type and culture medium on determining cancer selectivity of cold atmospheric plasma treatment. Cancers.

[16] Yan D., Cui H., Zhu W., Nourmohammadi N., Milberg J., Zhang L.G., Sherman J.H., Keidar M. (2017). The specific vulnerabilities of cancer cells to the cold atmospheric plasma-stimulated solutions. Sci. Rep..

[17] Van Loenhout J., Flieswasser T., Boullosa L.F., De Waele J., Van Audenaerde J., Marcq E., Jacobs J., Lin A., Lion E., Dewitte H. (2019). Cold atmospheric plasma-treated PBS eliminates immunosuppressive pancreatic stellate cells and induces immunogenic cell death of pancreatic cancer cells. Cancers.

[18] Boehm D., Heslin C., Cullen P.J., Bourke P. (2016). Cytotoxic and mutagenic potential of solutions exposed to cold atmospheric plasma. Sci. Rep..

[19] Labay C., Roldán M., Tampieri F., Stancampiano A., Bocanegra P.E., Ginebra M.P., Canal C. (2020). Enhanced generation of reactive species by cold plasma in gelatin solutions for selective cancer cell death. ACS Appl. Mater. Interfaces.

[20] Wang L., Yang X., Yang C., Gao J., Zhao Y., Cheng C., Zhao G., Liu S. (2019). The inhibition effect of cold atmospheric plasma-activated media in cutaneous squamous carcinoma cells. Future Oncol..

[21] Griseti E., Merbahi N., Golzio M. (2020). Anti-cancer potential of two plasma-activated liquids: Implication of long-lived reactive oxygen and nitrogen species. Cancers.

[22] Labay C., Hamouda I., Tampieri F., Ginebra M.P., Canal C. (2019). Production of reactive species in alginate hydrogels for cold atmospheric plasma-based therapies. Sci. Rep..

[23] Solé-Martí X., Espona-Noguera A., Ginebra M., Canal C. (2021). Plasma-conditioned liquids as anticancer therapies in vivo: Current state and future directions. Cancers.

[24] Lin A., Stapelmann K., Bogaerts A. (2020). Advances in plasma oncology toward clinical translation. Cancers.

[25] Freund E., Bekeschus S. (2021). Gas plasma-oxidized liquids for cancer treatment: Pre-clinical relevance, immuno-oncology, and clinical obstacles. IEEE Trans. Radiat. Plasma Med. Sci..

[26] Motaln H., Čerček U., Recek N., Česnik A.B., Mozetič M., Rogelj B. (2020). Cold atmospheric plasma induces stress granule formation via an eIF2α-dependent pathway. Biomater. Sci..

[27] Yadav D.K., Adhikari M., Kumar S., Ghimire B., Han I., Kim M.H., Choi E.H. (2020). Cold atmospheric plasma generated reactive species aided inhibitory effects on human melanoma cells: An in vitro and in silico study. Sci. Rep..

[28] Bauer G., Sersenová D., Graves D.B., Machala Z. (2019). Cold atmospheric plasma and plasma-activated medium trigger RONS-based tumor cell apoptosis. Sci. Rep..

[29] Tanaka H., Mizuno M., Katsumata Y., Ishikawa K., Kondo H., Hashizume H., Okazaki Y., Toyokuni S., Nakamura K., Yoshikawa N. (2019). Oxidative stress-dependent and -independent death of glioblastoma cells induced by non-thermal plasma-exposed solutions. Sci. Rep..

[30] Kondeti V.S.S.K., Phan C.Q., Wende K., Jablonowski H., Gangal U., Granick J.L., Hunter R.C., Bruggeman P.J. (2018). Long-lived and short-lived reactive species produced by a cold atmospheric pressure plasma jet for the inactivation of *Pseudomonas aeruginosa* and *Staphylococcus aureus*. Free Radic. Biol. Med..

[31] Gjika E., Pal-Ghosh S., Tang A., Kirschner M., Tadvalkar G., Canady J., Stepp M.A., Keidar M. (2018). Adaptation of Operational Parameters of Cold Atmospheric Plasma for in Vitro Treatment of Cancer Cells. ACS Appl. Mater. Interfaces.

[32] Golubitskaya E.A., Troitskaya O.S., Yelak E.V., Gugin P.P., Richter V.A., Schweigert I.V., Zakrevsky D.E., Koval O.A. (2019). Cold physical plasma decreases the viability of lung adenocarcinoma cells. Acta Nat..

[33] Alimohammadi M., Golpur M., Sohbatzadeh F., Hadavi S., Bekeschus S., Niaki H.A., Valadan R., Rafiei A. (2020). Cold atmospheric plasma is a potent tool to improve chemotherapy in melanoma in vitro and in vivo. Biomolecules.

[34] Wang M., Holmes B., Cheng X., Zhu W., Keidar M., Zhang L.G. (2013). Cold atmospheric plasma for selectively ablating metastatic breast cancer cells. PLoS ONE.

[35] Uchiyama H., Zhao Q.L., Hassan M.A., Andocs G., Nojima N., Takeda K., Ishikawa K., Hori M., Kondo T. (2015). EPR-spin trapping and flow cytometric studies of free radicals generated using cold atmospheric argon plasma and X-ray irradiation in aqueous solutions and intracellular milieu. PLoS ONE.

[36] Yan D., Talbot A., Nourmohammadi N., Cheng X., Canady J., Sherman J., Keidar M. (2015). Principles of using cold atmospheric plasma stimulated media for cancer treatment. Sci. Rep..

[37] Zubor P., Wang Y., Liskova A., Samec M., Koklesova L., Dankova Z., Dørum A., Kajo K., Dvorska D., Lucansky V. (2020). Cold atmospheric pressure plasma (CAP) as a new tool for the management of vulva cancer and vulvar premalignant lesions in gynaecological oncology. Int. J. Mol. Sci..

[38] Jo A., Joh H.M., Chung T.H., Chung J.W. (2020). Anticancer effects of plasma-activated medium produced by a microwave-excited atmospheric pressure argon plasma jet. Oxidative Med. Cell. Longev..

[39] Bauer G. (2016). The antitumor effect of singlet oxygen. Anticancer Res..

[40] Bauer G. (2018). Targeting protective catalase of tumor cells with cold atmospheric plasma- activated medium (PAM). Anticancer Agents Med. Chem..

[41] Yan D., Cui H., Zhu W., Talbot A., Zhang L.G., Sherman J.H., Keidar M. (2017). The strong cell-based hydrogen peroxide generation triggered by cold atmospheric plasma. Sci. Rep..

[42] Bauer G. (2019). The synergistic effect between hydrogen peroxide and nitrite, two long-lived molecular species from cold atmospheric plasma, triggers tumor cells to induce their own cell death. Redox Biol..

[43] Bauer G., Sersenová D., Graves D.B., Machala Z. (2019). Dynamics of singlet oxygen-triggered, RONS-based apoptosis induction after treatment of tumor cells with cold atmospheric plasma or plasma-activated medium. Sci. Rep..

[44] Bauer G. (2019). Intercellular singlet oxygen-mediated bystander signaling triggered by long-lived species of cold atmospheric plasma and plasma-activated medium. Redox Biol..

[45] Bengtson C., Bogaerts A. (2020). On the anti-cancer effect of cold atmospheric plasma and the possible role of catalase-dependent apoptotic pathways. Cells.

[46] Shaw P., Kumar N., Hammerschmid D., Privat-Maldonado A., Dewilde S., Bogaerts A. (2019). Synergistic effects of melittin and plasma treatment: A promising approach for cancer therapy. Cancers.

[47] Vijayarangan V., Delalande A., Dozias S., Pouvesle J.M., Robert E., Pichon C. (2020). New insights on molecular internalization and drug delivery following plasma jet exposures. Int. J. Pharm..

[48] Haralambiev L., Nitsch A., Jacoby J.M., Strakeljahn S., Bekeschus S., Mustea A., Ekkernkamp A., Stope M.B. (2020). Cold atmospheric plasma treatment of chondrosarcoma cells affects proliferation and cell membrane permeability. Int. J. Mol. Sci..

[49] Haralambiev L., Nitsch A., Einenkel R., Muzzio D.O., Gelbrich N., Burchardt M., Zygmunt M., Ekkernkamp A., Stope M.B., Gümbel D. (2020). The Effect of cold atmospheric plasma on the membrane permeability of human osteosarcoma cells. Anticancer Res..

[50] He Z., Liu K., Manaloto E., Casey A., Cribaro G.P., Byrne H.J., Tian F., Barcia C., Conway G.E., Cullen P.J. (2018). Cold atmospheric plasma induces ATP-dependent endocytosis of nanoparticles and synergistic U373MG cancer cell death. Sci. Rep..

[51] Jacoby J.M., Strakeljahn S., Nitsch A., Bekeschus S., Hinz P., Mustea A., Ekkernkamp A., Tzvetkov M.V., Haralambiev L., Stope M.B. (2020). An innovative therapeutic option for the treatment of skeletal sarcomas: Elimination of osteo- and ewing’s sarcoma cells using physical gas plasma. Int. J. Mol. Sci..

[52] Yan D., Talbot A., Nourmohammadi N., Sherman J.H., Cheng X., Keidar M. (2015). Toward understanding the selective anticancer capacity of cold atmospheric plasma—A model based on aquaporins. Biointerphases.

[53] Wang L.L., Qin S.B., Xu X.T., Hu C., Qian D.Q., Ye C., Zhou J.Y. (2016). Killing effect and its mechanism of low-temperature plasma on different human cancer cell lines. Chin. J. Oncol..

[54] Moniruzzaman R., Rehman M.U., Zhao Q.L., Jawaid P., Mitsuhashi Y., Imaue S., Fujiwara K., Ogawa R., Tomihara K., Saitoh J.I. (2018). Roles of intracellular and extracellular ROS formation in apoptosis induced by cold atmospheric helium plasma and X-irradiation in the presence of sulfasalazine. Free Radic. Biol. Med..

[55] Schneider C., Gebhardt L., Arndt S., Karrer S., Zimmermann J.L., Fischer M.J.M., Bosserhoff A.K. (2019). Acidification is an essential process of cold atmospheric plasma and promotes the anti-cancer effect on malignant melanoma cells. Cancers.

[56] Schneider C., Gebhardt L., Arndt S., Karrer S., Zimmermann J.L., Fischer M.J.M., Bosserhoff A.K. (2018). Cold atmospheric plasma causes a calcium influx in melanoma cells triggering CAP-induced senescence. Sci. Rep..

[57] Xu S., Wang Y., Que Y., Ma C., Cai S., Wang H., Yang X., Yang C., Cheng C., Zhao G. (2020). Cold atmospheric plasma activated Ringer’s solution inhibits the proliferation of osteosarcoma cells through the mitochondrial apoptosis pathway. Oncol. Rep..

[58] Murakami T. (2019). Numerical modelling of the effects of cold atmospheric plasma on mitochondrial redox homeostasis and energy metabolism. Sci. Rep..

[59] Adhikari M., Adhikari B., Ghimire B., Baboota S., Choi E.H. (2020). Cold atmospheric plasma and silymarin nanoemulsion activate autophagy in human melanoma cells. Int. J. Mol. Sci..

[60] Scharf C., Eymann C., Emicke P., Bernhardt J., Wilhelm M., Görries F., Winter J., von Woedtke T., Darm K., Daeschlein G. (2019). Improved wound healing of airway epithelial cells is mediated by cold atmospheric plasma: A time course-related proteome analysis. Oxidative Med. Cell. Longev..

[61] Arndt S., Unger P., Wacker E., Shimizu T., Heinlin J., Li J.F., Thomas H.M., Morfill G.E., Zimmermann J.L., Bosserhoff A.K. (2013). Cold atmospheric plasma (CAP) changes gene expression of key molecules of the wound healing machinery and improves wound healing in vitro and in vivo. PLoS ONE.

[62] Vaquero J., Judée F., Vallette M., Decauchy H., Arbelaiz A., Aoudjehane L., Scatton O., Gonzalez-Sanchez E., Merabtene F., Augustin J. (2020). Cold-atmospheric plasma induces tumor cell death in preclinical in vivo and in vitro models of human cholangiocarcinoma. Cancers.

[63] Judée F., Fongia C., Ducommun B., Yousfi M., Lobjois V., Merbahi N. (2016). Short and long time effects of low temperature Plasma Activated Media on 3D multicellular tumor spheroids. Sci. Rep..

[64] Hirst A.M., Simms M.S., Mann V.M., Maitland N.J., O’Connell D., Frame F.M. (2015). Low-temperature plasma treatment induces DNA damage leading to necrotic cell death in primary prostate epithelial cells. Br. J. Cancer.

[65] Schmidt A., Bekeschus S., Jarick K., Hasse S., von Woedtke T., Wende K. (2019). Cold physical plasma modulates p53 and mitogen-activated protein kinase signaling in keratinocytes. Oxidative Med. Cell. Longev..

[66] Shi L., Yu L., Zou F., Hu H., Liu K., Lin Z. (2017). Gene expression profiling and functional analysis reveals that p53 pathway-related gene expression is highly activated in cancer cells treated by cold atmospheric plasma-activated medium. PeerJ.

[67] Ji H.W., Kim H., Kim H.W., Yun S.H., Park J.E., Choi E.H., Kim S.J. (2020). Genome-wide comparison of the target genes of the reactive oxygen species and non-reactive oxygen species constituents of cold atmospheric plasma in cancer cells. Cancers.

[68] Yang F., Zhou Y., Yu H., Yang J., Zhu C., Ahmad N., Meng X., Zhao R., Zhuang J., Sun M. (2020). Combination of metformin and cold atmospheric plasma induces glioma cell death to associate with c-Fos. Neoplasma.

[69] Kim H.W., Jeong D., Ham J., Kim H., Ji H.W., Choi E.H., Kim S.J. (2020). ZNRD1 and its antisense long noncoding RNA ZNRD1-AS1 are oppositely regulated by cold atmospheric plasma in breast cancer cells. Oxidative Med. Cell. Longev..

[70] Li W., Yu H., Ding D., Chen Z., Wang Y., Wang S., Li X., Keidar M., Zhang W. (2019). Cold atmospheric plasma and iron oxide-based magnetic nanoparticles for synergetic lung cancer therapy. Free Radic. Biol. Med..

[71] Yang Y., Li D., Li Y., Jiang Q., Sun R., Liu J., Wu F., Miao J., Ni L., Shi X. (2020). Low-temperature plasma suppresses proliferation and induces apoptosis in lung cancer cells by regulating the miR-203a/BIRC5 axis. OncoTargets Ther..

[72] Lee S., Park S., Lee H., Jeong D., Ham J., Choi E.H., Kim S.J. (2018). ChIP-seq analysis reveals alteration of H3K4 trimethylation occupancy in cancer-related genes by cold atmospheric plasma. Free Radic. Biol. Med..

[73] Park S.B., Kim B., Bae H., Lee H., Lee S., Choi E.H., Kim S.J. (2015). Differential epigenetic effects of atmospheric cold plasma on MCF-7 and MDA-MB-231 breast cancer cells. PLoS ONE.

[74] Tornin J., Mateu-Sanz M., Rodríguez A., Labay C., Rodríguez R., Canal C. (2019). Pyruvate plays a main role in the antitumoral selectivity of cold atmospheric plasma in osteosarcoma. Sci. Rep..

[75] Xu D., Ning N., Xu Y., Wang B., Cui Q., Liu Z., Wang X., Liu D., Chen H., Kong M.G. (2019). Effect of cold atmospheric plasma treatment on the metabolites of human leukemia cells. Cancer Cell Int..

[76] Yang Y., Xu D., Ning N., Xu Y. (2019). Analysis of metabolite profiling in human endothelial cells after plasma jet treatment. Biomed Res. Int..

[77] Xu D., Xu Y., Ning N., Cui Q., Liu Z., Wang X., Liu D., Chen H., Kong M.G. (2018). Alteration of metabolite profiling by cold atmospheric plasma treatment in human myeloma cells. Cancer Cell Int..

[78] Ishikawa K., Hosoi Y., Tanaka H., Jiang L., Toyokuni S., Nakamura K., Kajiyama H., Kikkawa F., Mizuno M., Hori M. (2020). Non-thermal plasma-activated lactate solution kills U251SP glioblastoma cells in an innate reductive manner with altered metabolism. Arch. Biochem. Biophys..

[79] Haralambiev L., Bandyophadyay A., Suchy B., Weiss M., Kramer A., Bekeschus S., Ekkernkamp A., Mustea A., Kaderali L., Stope M.B. (2020). Determination of immediate vs. kinetic growth retardation in physically plasma-treated cells by experimental and modelling data. Anticancer Res..

[80] Verloy R., Privat-Maldonado A., Smits E., Bogaerts A. (2020). Cold atmospheric plasma treatment for pancreatic cancer—The importance of pancreatic stellate cells. Cancers.

[81] Gümbel D., Gelbrich N., Napp M., Daeschlein G., Kramer A., Sckell A., Burchardt M., Ekkernkamp A., Stope M.B. (2017). Peroxiredoxin expression of human osteosarcoma cells is influenced by cold atmospheric plasma treatment. Anticancer Res..

[82] Nakai N., Fujita R., Kawano F., Takahashi K., Ohira T., Shibaguchi T., Nakata K., Ohira Y. (2014). Retardation of C2C12 myoblast cell proliferation by exposure to low-temperature atmospheric plasma. J. Physiol. Sci..

[83] Volotskova T., Hawley T.S., Stepp M.A., Keidar M. (2012). Targeting the cancer cell cycle by cold atmospheric plasma. Sci. Rep..

[84] Siu A., Volotskova O., Cheng X., Khalsa S.S., Bian K., Murad F., Keidar M., Sherman J.H. (2015). Differential effects of cold atmospheric plasma in the treatment of malignant glioma. PLoS ONE.

[85] Privat-Maldonado A., Gorbanev Y., Dewilde S., Smits E., Bogaerts A. (2018). Reduction of human glioblastoma spheroids using cold atmospheric plasma: The combined effect of short- and long-lived reactive species. Cancers.

[86] Babington P., Rajjoub K., Canady J., Siu A., Keidar M., Sherman J.H. (2015). Use of cold atmospheric plasma in the treatment of cancer. Biointerphases.

[87] Stope M.B., Benouahi R., Sander C., Haralambiev L., Nitsch A., Egger E., Mustea A. (2020). Protherapeutic effects and inactivation of mammary carcinoma cells by a medical argon plasma device. Anticancer Res..

[88] Adhikari M., Kaushik N., Ghimire B., Adhikari B., Baboota S., Al-Khedhairy A.A., Wahab R., Lee S.J., Kaushik N., Choi E.H. (2019). Cold atmospheric plasma and silymarin nanoemulsion synergistically inhibits human melanoma tumorigenesis via targeting HGF/c-MET downstream pathway. Cell Commun. Signal..

[89] Kaushik N.K., Kaushik N., Wahab R., Bhartiya P., Linh N.N., Khan F., Al-Khedhairy A.A., Choi E.H. (2020). Cold atmospheric plasma and gold quantum dots exert dual cytotoxicity mediated by the cell receptor-activated apoptotic pathway in glioblastoma cells. Cancers.

[90] Tornín J., Villasante A., Solé-Martí X., Ginebra M., Canal C. (2021). Osteosarcoma tissue-engineered model challenges oxidative stress therapy revealing promoted cancer stem cell properties. Free Radic. Biol. Med..

[91] Gjika E., Pal-Ghosh S., Kirschner M.E., Lin L., Sherman J.H., Stepp M.A., Keidar M. (2020). Combination therapy of cold atmospheric plasma (CAP) with temozolomide in the treatment of U87MG glioblastoma cells. Sci. Rep..

[92] Xu D., Luo X., Xu Y., Cui Q., Yang Y., Liu D., Chen H., Kong M.G. (2016). The effects of cold atmospheric plasma on cell adhesion, differentiation, migration, apoptosis and drug sensitivity of multiple myeloma. Biochem. Biophys. Res. Commun..

[93] Chang C.H., Yano K.I., Sato T. (2020). Nanosecond pulsed current under plasma-producing conditions induces morphological alterations and stress fiber formation in human fibrosarcoma HT-1080 cells. Arch. Biochem. Biophys..

[94] Duchesne C., Banzet S., Lataillade J.J., Rousseau A., Frescaline N. (2019). Cold atmospheric plasma modulates endothelial nitric oxide synthase signalling and enhances burn wound neovascularisation. J. Pathol..

[95] Dezest M., Chavatte L., Bourdens M., Quinton D., Camus M., Garrigues L., Descargues P., Arbault S., Burlet-Schiltz O., Casteilla L. (2017). Mechanistic insights into the impact of cold atmospheric pressure plasma on human epithelial cell lines. Sci. Rep..

[96] Yoshikawa N., Liu W., Nakamura K., Yoshida K., Ikeda Y., Tanaka H., Mizuno M., Toyokuni S., Hori M., Kikkawa F. (2020). Plasma-activated medium promotes autophagic cell death along with alteration of the mTOR pathway. Sci. Rep..

[97] Conway G.E., He Z., Hutanu A.L., Cribaro G.P., Manaloto E., Casey A., Traynor D., Milosavljevic V., Howe O., Barcia C. (2019). Cold atmospheric plasma induces accumulation of lysosomes and caspase-independent cell death in U373MG glioblastoma multiforme cells. Sci. Rep..

[98] Arndt S., Wacker E., Li Y.F., Shimizu T., Thomas H.M., Morfill G.E., Karrer S., Zimmermann J.L., Bosserhoff A.K. (2013). Cold atmospheric plasma, a new strategy to induce senescence in melanoma cells. Exp. Dermatol..

[99] Bourdens M., Jeanson Y., Taurand M., Juin N., Carrière A., Clément F., Casteilla L., Bulteau A.L., Planat-Bénard V. (2019). Short exposure to cold atmospheric plasma induces senescence in human skin fibroblasts and adipose mesenchymal stromal cells. Sci. Rep..

[100] Yoon Y.J., Suh M.J., Lee H.Y., Lee H.J., Choi E.H., Moon I.S., Song K. (2018). Anti-tumor effects of cold atmospheric pressure plasma on vestibular schwannoma demonstrate its feasibility as an intra-operative adjuvant treatment. Free Radic. Biol. Med..

[101] Thiyagarajan M., Anderson H., Gonzales X.F. (2014). Induction of apoptosis in human myeloid leukemia cells by remote exposure of resistive barrier cold plasma. Biotechnol. Bioeng..

[102] Tabuchi Y., Uchiyama H., Zhao Q.L., Yunoki T., Andocs G., Nojima N., Takeda K., Ishikawa K., Hori M., Kondo T. (2016). Effects of nitrogen on the apoptosis of and changes in gene expression in human lymphoma U937 cells exposed to argon-based cold atmospheric pressure plasma. Int. J. Mol. Med..

[103] Xiang L., Xu X., Zhang S., Cai D., Dai X. (2018). Cold atmospheric plasma conveys selectivity on triple negative breast cancer cells both in vitro and in vivo. Free Radic. Biol. Med..

[104] Yang X., Yang C., Wang L., Cao Z., Wang Y., Cheng C., Zhao G., Zhao Y. (2020). Inhibition of basal cell carcinoma cells by cold atmospheric plasma activated solution and differential gene expression analysis. Int. J. Oncol..

[105] Xia J., Zeng W., Xia Y., Wang B., Xu D., Liu D., Kong M.G., Dong Y. (2019). Cold atmospheric plasma induces apoptosis of melanoma cells via Sestrin2-mediated nitric oxide synthase signaling. J. Biophotonics.

[106] Xu D., Xu Y., Cui Q., Liu D., Liu Z., Wang X., Yang Y., Feng M., Liang R., Chen H. (2018). Cold atmospheric plasma as a potential tool for multiple myeloma treatment. Oncotarget.

[107] Turrini E., Laurita R., Stancampiano A., Catanzaro E., Calcabrini C., Maffei F., Gherardi M., Colombo V., Fimognari C. (2017). Cold atmospheric plasma induces apoptosis and oxidative stress pathway regulation in T-lymphoblastoid leukemia cells. Oxidative Med. Cell. Longev..

[108] Weiss M., Gümbel D., Gelbrich N., Brandenburg L.O., Mandelkow R., Zimmermann U., Ziegler P., Burchardt M., Stope M.B. (2015). Inhibition of cell growth of the prostate cancer cell model LNCaP by cold atmospheric plasma. Vivo.

[109] Weiss M., Gümbel D., Hanschmann E.M., Mandelkow R., Gelbrich N., Zimmermann U., Walther R., Ekkernkamp A., Sckell A., Kramer A. (2015). Cold atmospheric plasma treatment induces anti-proliferative effects in prostate cancer cells by redox and apoptotic signaling pathways. PLoS ONE.

[110] Gümbel D., Gelbrich N., Weiss M., Napp M., Daeschlein G., Sckell A., Ender S.A., Kramer A., Burchardt M., Ekkernkamp A. (2016). New treatment options for osteosarcoma—Inactivation of osteosarcoma cells by cold atmospheric plasma. Anticancer Res..

[111] Schneider C., Arndt S., Zimmermann J.L., Li Y., Karrer S., Bosserhoff A.K. (2018). Cold atmospheric plasma treatment inhibits growth in colorectal cancer cells. Biol. Chem..

[112] Kurita H., Haruta N., Uchihashi Y., Seto T., Takashima K. (2020). Strand breaks and chemical modification of intracellular DNA induced by cold atmospheric pressure plasma irradiation. PLoS ONE.

[113] Haralambiev L., Neuffer O., Nitsch A., Kross N.C., Bekeschus S., Hinz P., Mustea A., Ekkernkamp A., Gümbel D., Stope M.B. (2020). Inhibition of angiogenesis by treatment with cold atmospheric plasma as a promising therapeutic approach in oncology. Int. J. Mol. Sci..

[114] Yang X., Chen G., Yu K.N., Yang M., Peng S., Ma J., Qin F., Cao W., Cui S., Nie L. (2020). Cold atmospheric plasma induces GSDME-dependent pyroptotic signaling pathway via ROS generation in tumor cells. Cell Death Dis..

[115] Kroemer G., Galluzzi L., Kepp O., Zitvogel L. (2013). Immunogenic cell death in cancer therapy. Annu. Rev. Immunol..

[116] Alzeibak R., Mishchenko T.A., Shilyagina N.Y., Balalaeva I.V., Vedunova M.V., Krysko D.V. (2021). Targeting immunogenic cancer cell death by photodynamic therapy: Past, present and future. J. Immunother. Cancer.

[117] Azzariti A., Iacobazzi R.M., Di Fonte R., Porcelli L., Gristina R., Favia P., Fracassi F., Trizio I., Silvestris N., Guida G. (2019). Plasma-activated medium triggers cell death and the presentation of immune activating danger signals in melanoma and pancreatic cancer cells. Sci. Rep..

[118] Troitskaya O., Golubitskaya E., Biryukov M., Varlamov M., Gugin P., Milakhina E., Richter V., Schweigert I., Zakrevsky D., Koval O. (2020). Non-thermal plasma application in tumor-bearing mice induces increase of serum HMGB1. Int. J. Mol. Sci..

[119] Yoon Y., Ku B., Lee K., Jung Y.J., Baek S.J. (2019). Cold atmospheric plasma induces HMGB1 expression in cancer cells. Anticancer Res..

[120] Arndt S., Landthaler M., Zimmermann J.L., Unger P., Wacker E., Shimizu T., Li Y.F., Morfill G.E., Bosserhoff A.K., Karrer S. (2015). Effects of cold atmospheric plasma (CAP) on β-defensins, inflammatory cytokines, and apoptosis-related molecules in keratinocytes in vitro and in vivo. PLoS ONE.

